# COVID-19 sentiment analysis via deep learning during the rise of novel cases

**DOI:** 10.1371/journal.pone.0255615

**Published:** 2021-08-19

**Authors:** Rohitash Chandra, Aswin Krishna

**Affiliations:** 1 School of Mathematics and Statistics, University of New South Wales, Sydney, Australia; 2 Department of Chemical Engineering, Indian Institute of Technology Guwahati, Guwahati, Assam, India; Bucharest University of Economic Studies, ROMANIA

## Abstract

Social scientists and psychologists take interest in understanding how people express emotions and sentiments when dealing with catastrophic events such as natural disasters, political unrest, and terrorism. The COVID-19 pandemic is a catastrophic event that has raised a number of psychological issues such as depression given abrupt social changes and lack of employment. Advancements of deep learning-based language models have been promising for sentiment analysis with data from social networks such as Twitter. Given the situation with COVID-19 pandemic, different countries had different peaks where rise and fall of new cases affected lock-downs which directly affected the economy and employment. During the rise of COVID-19 cases with stricter lock-downs, people have been expressing their sentiments in social media. This can provide a deep understanding of human psychology during catastrophic events. In this paper, we present a framework that employs deep learning-based language models via long short-term memory (LSTM) recurrent neural networks for sentiment analysis during the rise of novel COVID-19 cases in India. The framework features LSTM language model with a global vector embedding and state-of-art BERT language model. We review the sentiments expressed for selective months in 2020 which covers the major peak of novel cases in India. Our framework utilises multi-label sentiment classification where more than one sentiment can be expressed at once. Our results indicate that the majority of the tweets have been positive with high levels of optimism during the rise of the novel COVID-19 cases and the number of tweets significantly lowered towards the peak. We find that the optimistic, annoyed and joking tweets mostly dominate the monthly tweets with much lower portion of negative sentiments. The predictions generally indicate that although the majority have been optimistic, a significant group of population has been annoyed towards the way the pandemic was handled by the authorities.

## 1 Introduction

There has been unprecedented growth of information via social media which has been of interest to social scientists and psychologists in a better understanding of the human condition, psychology [1–4] and mental health [5]. Social media platforms such as Twitter has been used as a medium for data acquisition for research in psychology and behavioural science [6]. It has also been used as a tool for predicting personality type [1, 2], and understanding trends and backgrounds of users online [7, 8]. There has also been interest regarding how people express sentiments when dealing with catastrophic events such as natural disasters, extreme political viewpoints [9], and terrorism [10]. For instance, in the case of a terror attack in Kenya [10], Twitter became a crucial channel of communication between the government, emergency response team and the public.

Sentiment analysis involves the use of natural language processing (NLP) [11] methods to systematically study affective states and emotion understanding of individuals or social groups [12–14]. We note that deep learning, which is a machine learning method, has been prominently used for NLP tasks. Apart from research in psychology, sentiment analysis has a number of applications such as understanding customer behaviour [15], clinical medicine [16], building better prediction model for trading stocks [17], and elections such as the US Presidential campaign in 2012 [18]. Recently, there has been a trend of using deep learning-based language models [19] with training data from Twitter for sentiment analysis [20, 21]. One of the earliest works began using NLP methods such as n-grams with hash tags for building training data and machine learning methods such as Adaboost for sentiment classification [20]. Deep learning methods such as convolutional neural networks have been used for sentiment analysis on Twitter [22].

The *coronavirus disease 2019* (COVID-19) [23–26] global pandemic has been a catastrophic event with major impact on the world’s economy which created rise in unemployment, psychological issues, and depression. The abrupt social, economic and travel changes has motivated research from various fields [27–29], where computational modelling with machine learning has been prominent [30]; however, it had a number of challenges due to the testing and reporting of cases [31]. Deep learning models have played a significant role in forecasting COVID-19 infection treads for various parts of the world [32–34].

During rise of COVID-19 cases, and stricter lock downs, people have been expressing different sentiments in social media such as Twitter. Social media has played a significant role during COVID-19 which has driven researchers for analysis with NLP and machine learning methods. A study that used sentiment analysis via deep learning reported that World Health Organisation (WHO) tweets have been unsuccessful in providing public guidance [35]. There has been a study on sentiment analysis to study the effect of nationwide lockdown due to COVID-19 outbreak in India where it was found that people took the fight against COVID19 positively and majority were in agreement with the government for the initial nation-wide lockdown [36]. Social media posts and tweets brings another level of understanding when combined with sentiment analysis. An example of topic modelling examined tweets during the COVID-19 pandemic identified themes such as ‘origin of the virus’, and ‘the economy’ [37]. Topic modelling has been used with Twitter based sentiment analysis during early stages of COVID-19 and sentiments such as fear was dominant [38]. In region specific studies, tweets from the United States was used to determine the network of dominant topics and sentiments [39]. Further work was done in case of China via *bi-directional encoder representations from transformers* (BERT) language model for trend, topic, and sentiment analysis [40]. Further region specific studies include community sentiment analysis in Australia [41] and sentiment analysis in Nepal [42], where majority positive sentiments were found with elements of fear. In Spain, sentiment analysis reviewed how digital platforms created an impact during COVID-19 [43]. A study of cross-language sentiment analysis of European Twitter messages during the first few months of the COVID-19 pandemic found that the lockdown announcements correlate with a deterioration of moods, which recovers within a short time span [44].

The sentiment analysis examples from Europe and India show how social media can play a powerful role in understanding the psychology and the human condition Recent advancements of deep learning models as a tool for building robust language models have provided further motivation in understanding the temporal nature of the sentiments during and after the first peak of COVID-19 in India. Different countries had different peaks where rise and fall of new cases implemented lock-downs which directly affected the economy and employment. India is special in this way (until February 2021), where a single nation-wide peak was seen and only certain states had multiple peaks (Delhi and Maharashtra) [33].

In this paper, we use deep learning based language models via long short-term memory (LSTM) recurrent neural networks for sentiment analysis via tweets with a focus of rise of novel cases in India. We use LSTM and bidirectional LSTM (BD-LSTM) model with global vector (GloVe) for word representation for building a language model. Moreover, we use the BERT model to compare the results from LSTM and BD-LSTM models and then use the best model for COVID-19 sentiment analysis for the case of India. We use three datasets, which include India, along with the state of Maharashtra (includes Mumbai) and Delhi. We compare the monthly sentiments expressed covering the major peak of new cases in 2020. We present a framework that focuses on multi-label sentiment classification, where more than one sentiment can be expressed at once. We use Senwave COVID-19 sentiment dataset [45] which features 10,000 tweets collected worldwide and hand-labelled by 50 experts for training LSTM models.

We highlight that there is no study that uses language models for sentiment analysis during the rise of novel COVID-19 cases. Our framework compares the different types of sentiments expressed across the different months in relation to the rise of the number of cases, which impacted the economy and had different levels of lock downs. This had an effect on the psychology of the population given stress and fear. Hence, the study is a way to quantify and validate emotional and psychological conditions given uncertainty about the pandemic. The major contribution of the paper is in using state-of-art sentiment analysis methods for understanding the public behaviour in terms of psychological well being in relation to the rise of novel COVID-19 infections. This study presents a novel framework that makes use of information from social media for understanding public behavior during a major disruptive event of the century.

The rest of the paper is organised as follows: Section 2 presents a background of related work, and Section 3 presents the proposed methodology with data analysis. Section 4 presents experiments and results. Section 5 provides a discussion and Section 6 concludes the paper with discussion of future work.

## 2 Related work

Word embedding is the process of feature extraction from text for NLP tasks such as sentiment analysis [46–48]. Word embedding can be obtained using methods where words or phrases from the vocabulary are mapped to vectors of real numbers. The process generally involves a mathematical embedding from a large corpus with many dimensions per word to a vector space with a lower dimension that is useful for machine learning or deep learning models for text classification tasks [46]. Basic word embedding methods such as *bag of words* [49] and *term frequency inverse document frequency* [50] do not have context awareness and semantic information in embedding. This is also a problem for skip-grams [51] that use n-grams (such as bigrams and tri-grams) to develop word embedding, and in addition allow adjacent sequences of words tokens to be “skipped” [52].

Over the last decade, there has been phenomenal progress in the area of world embedding and language models. Mikolov et al. [53] proposed *word2vec* embedding which uses a feedforward neural network model to learn word associations from a text dataset which can detect synonymous words or suggest additional words given a partial sentence. It uses continuous bag-of-words (CBOW) or continuous skip-gram model architectures to produce a distributed representation of words. The method is used to create a large vector which represent each unique word in the corpus where semantic information and relation between the words are preserved. It has been shown that for two sentences that do not have much words in common, their semantic similarity can be captured using word2vec [53]. The limitation of word2vec is that it does not well represent the context of a word. Pennington et al. [54] for obtaining vector representations for words by mapping words into a meaningful space where the distance between words is related to semantic similarity. GloVe uses matrix factorization to constructs a large matrix of co-occurrence information to obtain representation that showcase linear substructures of the word vector space. The embedding feature vectors with top list words that match with certain distance measures. GloVe can be used to find relations between words such as synonyms, company-product relations. Due to the awareness in ethics in machine learning, there has been a major focus on ethical issues in NLP. A recent study showed that GloVe can have gender biased information; hence, a gender neutral GloVe method has been proposed [55].

There are some studies that review the effectiveness of word embedding methods. Ghannay et al. [56] provided an evaluation of word embedding methods such as GloVe [54], skip-gram, and continuous space language models (CSLM) [57]. The authors reported that skip-gram and GloVe outperformed CSLM in all the language tasks. Wang et al. [58] evaluated word embedding methods such as GloVe for applications of biomedical text analysis where it was found that word embedding trained from clinical notes and literature better captured word semantics.

## 3 Methodology

### 3.1 LSTM and BERT language models

Recurrent neural networks (RNN) in general feature a context memory layer to incorporate previous state and current inputs for propagating information to the next state, and eventually the output layer for decision making. Canonical RNNs (simple RNNs) feature several different architectures, besides the Elman RNN [59, 60] for modelling temporal sequences and dynamical systems [61–63]. One of the major challenges in training simple RNNs is due to the architectures properties of unfolding in time for long-term dependency problems. Backpropagation through time (BPTT), which is an extension of the backpropagation algorithm, has been prominent for training simple RNNs [64]. Due to problem of learning long-term dependencies given vanishing and exploding gradients with simple RNNs [65], long short-term memory (LSTM) recurrent neural networks have been developed [66]. LSTM networks have better capabilities for learning long-term dependencies in temporal data using memory cells and gates.

In the last decade, with the deep learning revolution, several LSTM architectures have been developed. Bidirectional LSTM models [67] process information in two directions, rather than making use of only previous context state for determining the next states which are based on bidirectional RNNs [68]. In this way, two independent LSTM models allow both backward and forward information about the sequence at every time step. This enables better access to long range state information which have been useful for word embedding [67] and several other sequence processing problems [67, 69, 70].

A transformer is an extended LSTM model that adopts the mechanism of attention which mimics cognitive attention to enhance important parts of the data while fading the rest [71]. Transformers also use an encoder-decoder architecture and have mostly been used for NLP tasks such as translation and text summarising [71, 72]. In comparison to conventional RNNs, transformers do not require data to be processed in a sequential order since the attention operation provides context for any position in the input sequence. BERT is a pre-trained transformer-based model for NLP tasks which has been used to better understand user behaviour in Google search engine [73]. BERT can be used for a number of other NLP applications such as clinical data processing [74]. The original BERT [73] has two models: 1.) BERT-base features 12 encoders with 12 bidirectional self-attention heads, and 2.) BERT-large features 24 encoders with 16 bidirectional self-attention heads. These are pre-trained from unlabeled data extracted from a corpus with 800 million words and English Wikipedia with 2,500 million words, respectively. Word2vec and GloVe are content-free models that generate a single word embedding representation for each word, whereas BERT takes into account the context for each occurrence of a given word which makes BERT one of the best language models. Hence, BERT is suitable for our sentiment analysis tasks using tweets during rise of COVID-19 cases in India.

### 3.2 Twitter-based sentiment analysis framework for COVID-19

**Fig 1:**
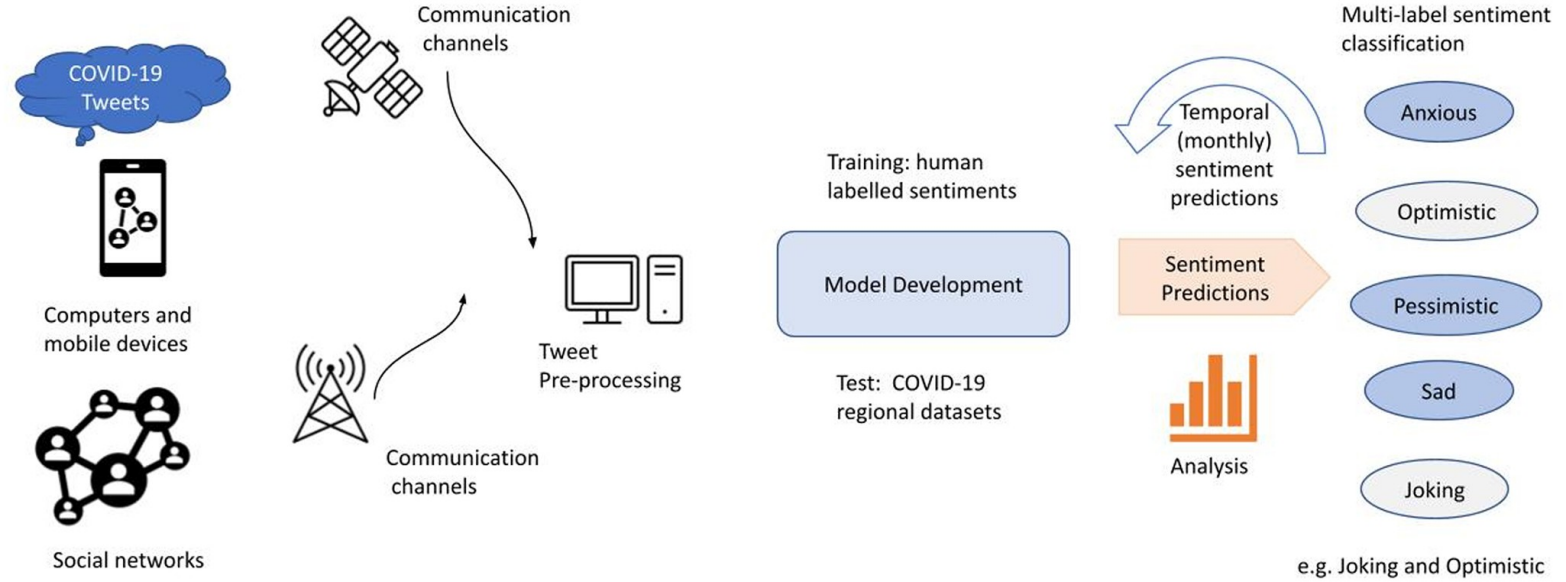
Framework: Twitter-based sentiment analysis for COVID-19 with model development via LSTM, BD-LSTM and BERT. After training, the best model is chosen for prediction of COVID-19 tweets from India. Note that the framework features multi-label classification which has the ability to provide more than two outcomes at once; i.e. a tweet can be optimistic and joking at the same time.

Our framework for sentiment analysis with deep learning follows a series of steps with major components that involve: 1.) tweet extraction; 2.) tweet pre-processing; 3.) model development and training using LSTM, BD-LSTM and BERT; and 4.) prediction using selected COVID-19 data. Fig 1 shows the framework diagram where we highlight the major components. We note that the framework features multi-label classification which has multiple outcomes at once, i.e the tweet can be optimistic and joking at the same time.

The first step involves processing on COVID-19 dataset from a selected region. We choose COVID-19 tweets from while of India along with two states which have been amongst the highest states of COVID-19 cases in 2020 (Maharashtra and Delhi). We process Twitter data obtained for India and related state as follows. We note that special phrases, emotion symbols (emoji’s) and abbreviations are present in tweets, since language in social media has been rapidly evolving. Hence, we have to transform them for building the language model. Table 1 We note that since this is a case of India, where English is used in combination with some key indigenous Indian languages (such as Hindi). Hence, we transform certain words, emotions and character symbols expressed in other languages as well. Note that we use hand-labelled sentiment dataset (Senwave COVID-19 dataset) [45] which features 11 different sentiments labelled by a group of 50 experts for 10,000 tweets worldwide during COVID-19 pandemic in 2020. In this dataset, “official report” is classified as a sentiment although it is a topic.

**Table 1:** Examples of word transformation that features changing symbols and emoji’s to words that can have semantic representation.

Original Phrase/Emoji	Transformed Word
omg	oh my god
btw	by the way
socialdistancing	social distancing
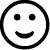	smiling face
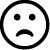	sad face
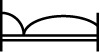	bed
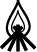	fire
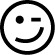	wink
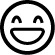	laugh

The next step involves converting each word into its corresponding GLoVe embedding vector, where each word is converted into a vector (300 dimensions in our case). We select GLoVe embedding since it has shown good results for language models with sentiment analysis in the literature [54]. The aforementioned vector from each word thus obtained is passed on to the respective LSTM models for training once the model architecture has been defined as shown in Fig 1. We first evaluate our trained models (LSTM, BD-LSTM, and BERT) and then use it for COVID-19 sentiment analysis using test data (India, Maharashtra, and Delhi). As shown in Fig 1, the trained model is used to classify 11 sentiments such as “anxious”, “sad”, and “joking”.

It is essential to have the right metric to evaluate the model for the given application. The outcome of a misclassification in a multi-label classification is no longer a correct or an incorrect instance as opposed to binary classification or multi-class classification [75]. In multi-label classification, a prediction that features a subset of the actual classes are considered better than a prediction that contains none of the actual classes; hence, this needs to be captured by the metric that captures the loss or gives a score. As given in the literature [75], multi-label classification evaluation is typically based on 1.) binary cross-entropy (BCE) loss [76] which is a softmax activation combined with a cross-entropy loss; 2.) Hamming loss [75] which calculates loss generated in the bit string of class labels using *exclusive or* (XOR) operation between the actual and predicted labels and then computes the average over the instances of the dataset. The Jaccard coefficient score [77] provides a measure of the overlap between actual and predicted labels with their attributes capturing similarity and diversity. Furthermore, label ranking average precision (LRAP) score [78] finds the percentage of the higher-ranked labels that resemble true labels for each of the given samples. F1-score conveys the balance between the precision and the recall. F1-score has been prominent for understanding class imbalance problems [79]. In application to multi-label classification, two types of F1-scores are predominantly used. F1-macro is computed using the F1-score per class/label and then averaging them, whereas F1-micro computes the F1-score for the entire data considering the total true positives, false negatives and false positives [80]. We use a combination of these scores to evaluate our model results for the first phase of the framework where the respective models are trained.

### 3.3 Model training

We present an experimental study that compares multi-label classification using LSTM, BD-LSTM, and BERT models as shown in the framework (Fig 1). We use 90% of the dataset for training and 10% for testing. We use the respective models and train them using the Senwave COVID-19 dataset which is publicly available, but restricted and needs permission from the authors for usage [45]. Therefore, we cannot provide the processed dataset, but rather provide the trained models via Github repository (https://github.com/sydney-machine-learning/COVID19_sentinentanalysis). The Senwave dataset features 10,000 Tweets collected from March to May 2020.

In the LSTM model, we determine the model hyper-parameters based on how the model performs in trial experiments. The GloVe embedding uses a word vector of size 300 size to provide data representation which is a standard representation in the literature [54]. We use a dropout regularisation probability of 0.65 for LSTM and BD-LSTM models which feature: 300 input units, two layers with 128 and 64 hidden units, and finally an output layer with 11 units for classifying the sentiments. In the case of BERT, we use the default hyper-parameters for the BERT-base (uncased) model and only tune the learning rate. The only major addition is a dropout layer at the end of the BERT architecture followed by a linear activation layer with 11 outputs corresponding to the 11 sentiments.

Table 2 shows the model training results (mean) for 10 experiments with different initial weights and biases for the respective models with different performance metrics. Generally, we find that BERT is better than LSTM and BD-LSTM models across the different performance metrics shown in Table 2. Hence, we proceed with the BERT model for evaluating the test dataset with a visualization of the predictions.

**Table 2:** Training performance for BD-LSTM, LSTM and BERT model using Senwave COVID-19 training dataset. Note that except for the BCE and Hamming loss, higher scores shows better performance.

Metric	BD-LSTM	LSTM	BERT
BCE Loss	0.281	0.255	0.372
Hamming Loss	0.163	0.157	0.142
Jaccard Score	0.417	0.418	0.510
LRAP Score	0.503	0.511	0.766
F1 Macro	0.434	0.430	0.530
F1 Micro	0.495	0.493	0.587

### 3.4 Application to COVID-19 pandemic in India

India COVID-19 pandemic management had major challenges given large population with a large number of densely populated cities [81]. India reported first COVID-19 case on 30th January 2020 and entered a lock-down afterwards which was gradually changed. India (22-nd March, 2021) had more than 11.6 million confirmed cases with more than 160 thousand deaths which made India the third highest confirmed cases in the world after the United States and Brazil. India was the 8th in world with more than 300 thousand active cases (prior to the second wave). India had a single major peak around middle of September in 2020 with close to 100 thousand cases daily, which gradually decreased to around 11 thousand cases daily end of January 2021, and since then it was gradually raising. In March 2021, the cases began rising faster and by 22nd March, India had 47 thousand daily new cases which was moving towards a second peak [82].

In the first six months, the states of Maharashtra (population of about 124 million), Delhi (population of 19 million), and Tamil Nadu (population of about 8 million) led COVID-19 infections [83]. Note that the city of Mumbai is in the state of Maharashtra, which in terms of the population can be compared to some of the highly populated countries. In the later half of the year, Delhi cases reduced but still remained in leading 8 states [33]. In 2021, Maharashtra continued as state of highest infections and in March, it was featuring more than half of new cases on a weekly basis and Delhi contained the situation with less than a thousand daily cases. Hence, our focus is to study whole of India with two states of interest that includes Maharashtra and Delhi.

**Fig 2:**
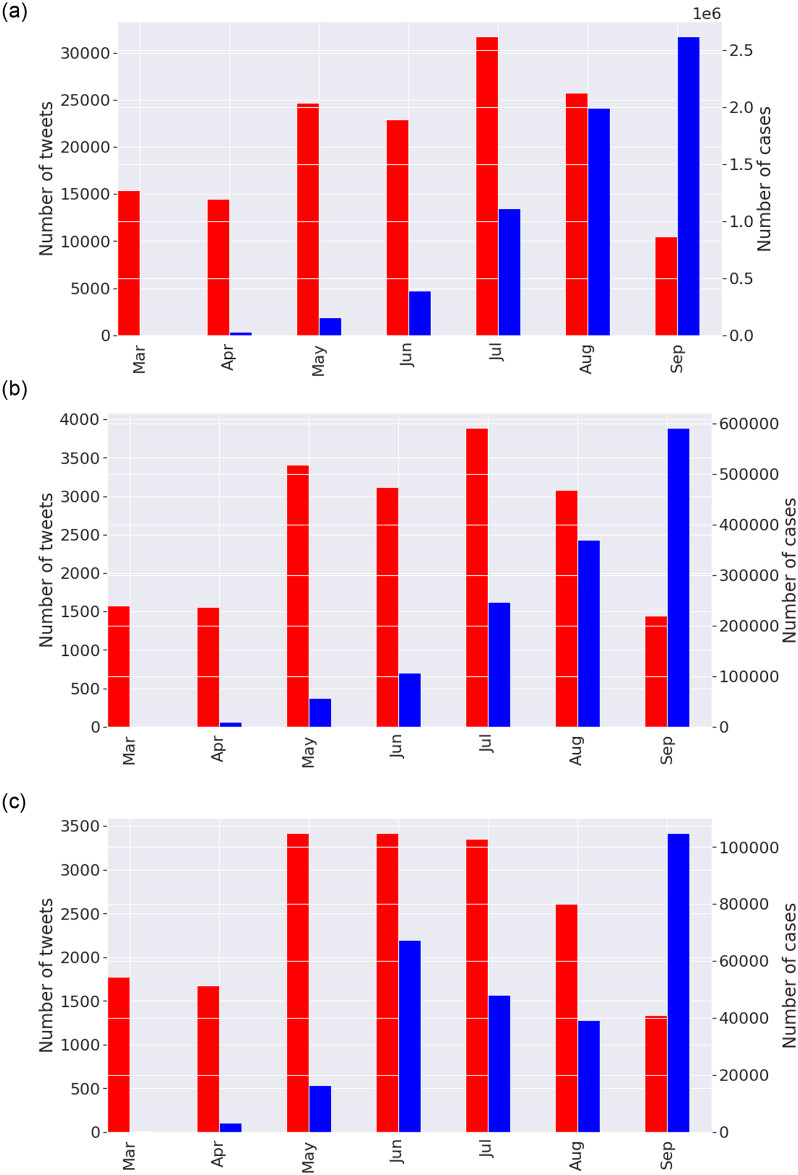
Novel COVID-19 tweets and cases in India, Maharashtra, and Delhi. The red bars indicate the number of tweets while the black bars indicate the number of novel cases.

We note that the proposed framework can be applied to any part of the world; however, we are choosing the case of India to show the effectiveness of the framework. The final step is in applying the different datasets from COVID-19 in India which include, nation-wide COVID-19 sentiments, and two major states with COVID-19 cases. The trend in the cases shows that both states had a major peak followed by minor ones, whereas India as a whole had a single major peak which was around mid-September 2020 with close to 97,000 novel cases per day during the peak as shown in the next section (Fig 2).

## 4 Results

In this section, we provide results of the implementation of our proposed framework using Twitter dataset for COVID-19 in India.

### 4.1 COVID-19 data visualisation

Our test dataset [84] features COVID-19 India which features tweets associated with COVID-19 from March to September 2020. It consists of more than 150,000 tweets from India. We generate two other datasets from this which feature the regions of Maharashtra (state) and Delhi (union territory) which contains around 18,000 tweets each, respectively.

We first provide visualisation of the dataset and report features of interest to language models such as bi-grams, and tri-grams and distribution of monthly tweets. Fig 2 shows the distribution of number of tweets for selected months along with the number of cases for the respective datasets.

We notice that the number of tweets in the case of India follows the same trend as the number of novel COVID-19 cases until July, afterwards the number of tweets decline (Panel a, Fig 2). There is a similar pattern for case of Maharashtra (Panel b). The case of Delhi (Panel c) is slightly different as the first peak was reached in July with increasing tweets that declined afterwards and did not keep up with the second peak of cases in September. This indicates that as more cases were observed in the early months, there was much concern which eased before the major peak was reached and the number of tweets were drastically decreased. There could be elements of fear, depression and anxiety as the tweets decreased drastically after July with increasing cases, which we analyse in the following section.

**Fig 3:**
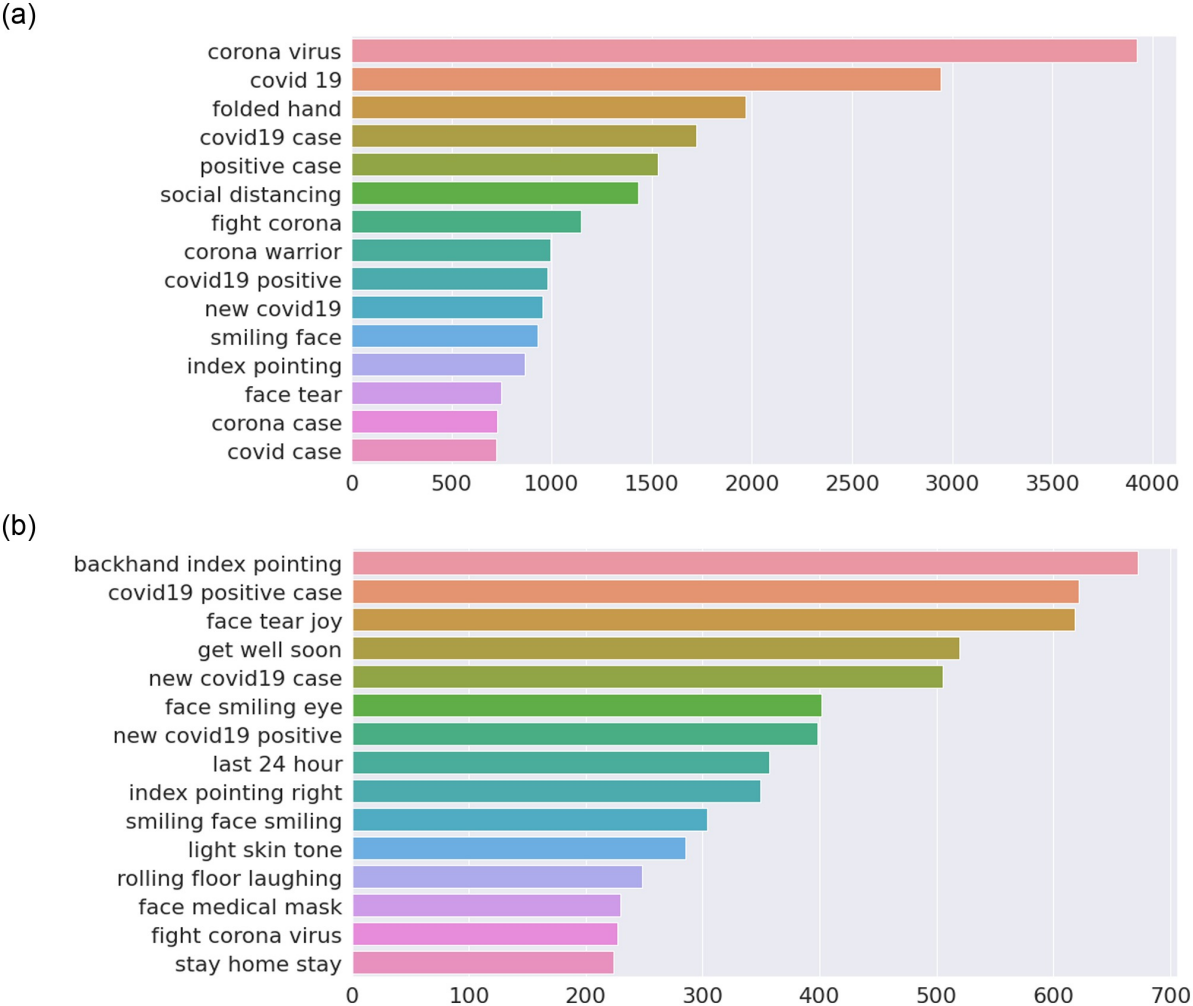
Bi-grams and tri-grams for case of India. Note that a combination of emoji’s such as “backhand—index—pointing”, “smiling—face”, and words have been used. (a) Bi-grams. (b) Tri-grams.

Fig 3 shows a visualisation of the number of bi-grams and tri-grams for the case of India. In case of bi-grams (Panel a), we find that the term “corona—virus” is mostly used followed by “covid—19” which are essentially the same. Next, it is interesting that the terms “folded—hand” are mostly used followed by “positive—case” and “social—distancing”. The term “folded—hand” shows Indian social and religious symbol that denotes keeping the faith, giving respect and also a sign of acknowledgement and appreciation. We note that the “folded hand” is an emotion icon (emoji) used predominantly in social media, and hence been processed as a text during prepossessing (Table 1), which is actually is not a bi-gram from a semantic viewpoint. In order to provide better understanding of the context of the tweets, we give examples in Table 3. In the case of tri-grams (Panel b), we find more information in tweets such as “backhand—index—pointing” which is an emoji; hence, we provide some of the tweets that fall in this category in Table 4.

**Table 3:** Selected examples of processed tweets that are captured in most prominent bi-grams.

Month	Tweet	Bi-gram (Emoji)
March	“releasing today at 6:00 pm on my youtube channel! let’s fight this together folded hands i need your support guys”	“folded hand”
July	“that’s really shameful and heinous folded hands”	“folded hand”
August	“please applaud this corona economy warrior. folded hands kudos.”	“folded hand”
March	“india this backhand index pointing down”	“index pointing”
May	“corona: to everyone backhand index pointing down”	“index pointing”
July	“backhand index pointing right. ……‥lockdown time…‥ #picture #instagood #instadaily #insta”	“index pointing”

**Table 4:** Selected examples of processed tweets that are captured in most prominent tri-grams.

Month	Tweet	Tri-gram (Emoji)
May	“google doc of resources for amphan and covid. backhand index pointing down. retweet for greater reach.”	“backhand—index—pointing”
August	“#covid tips backhand index pointing down”	“backhand—index—pointing”
September	“backhand index pointing right registers highest single-day cases in the world—95,529”	“backhand—index—pointing”

**Fig 4:**
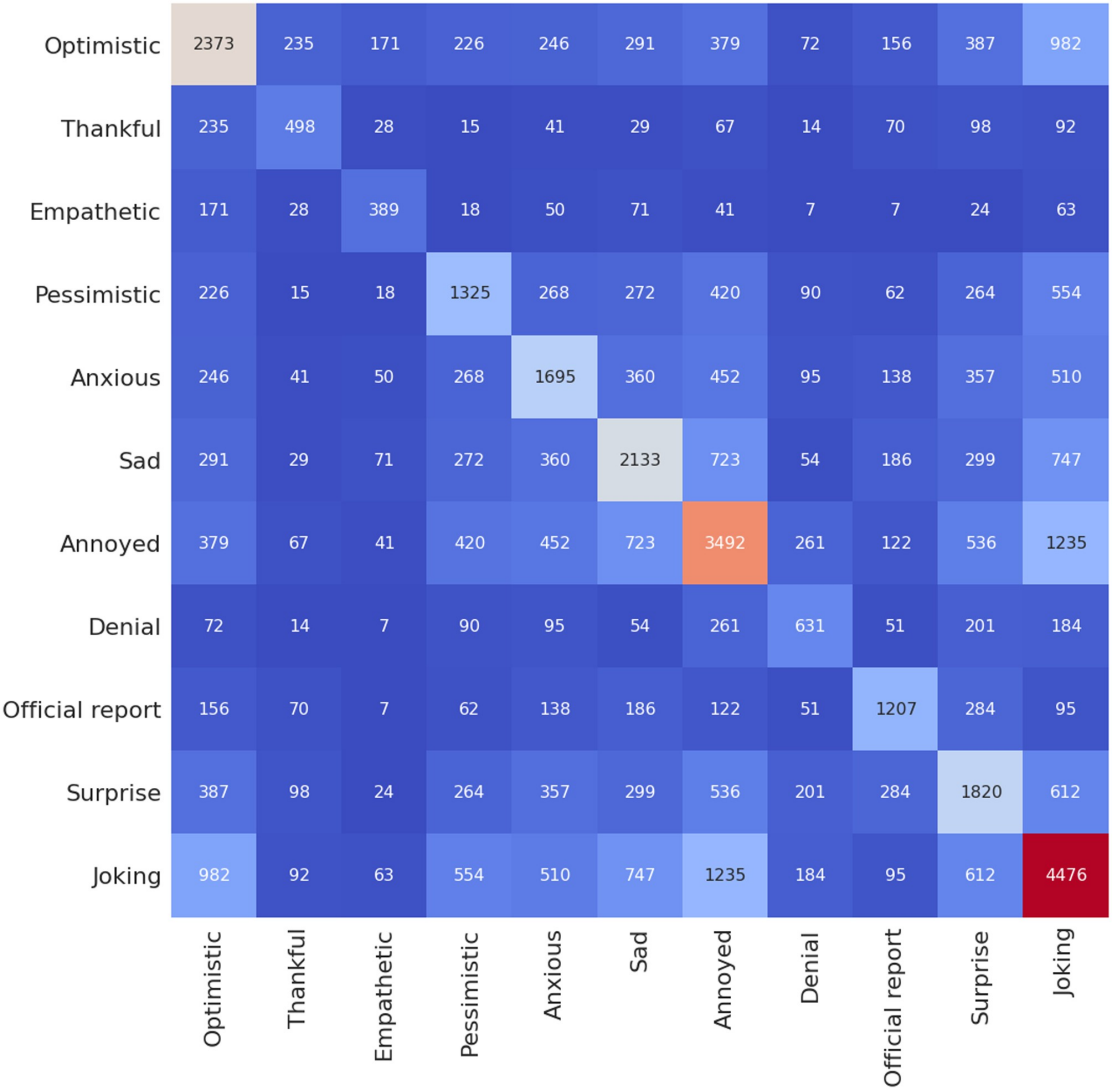
Heatmap showing number of occurrence of a given sentiment in relation to the rest of the sentiments for 10,000 tweets from Senwave dataset [45] used for training.

Fig 4 shows number of occurrence of a given sentiment in relation to the rest of the sentiments for 10,000 hand-labelled tweets in Senwave dataset [45] used for training.

### 4.2 Sentiment prediction

**Fig 5:**
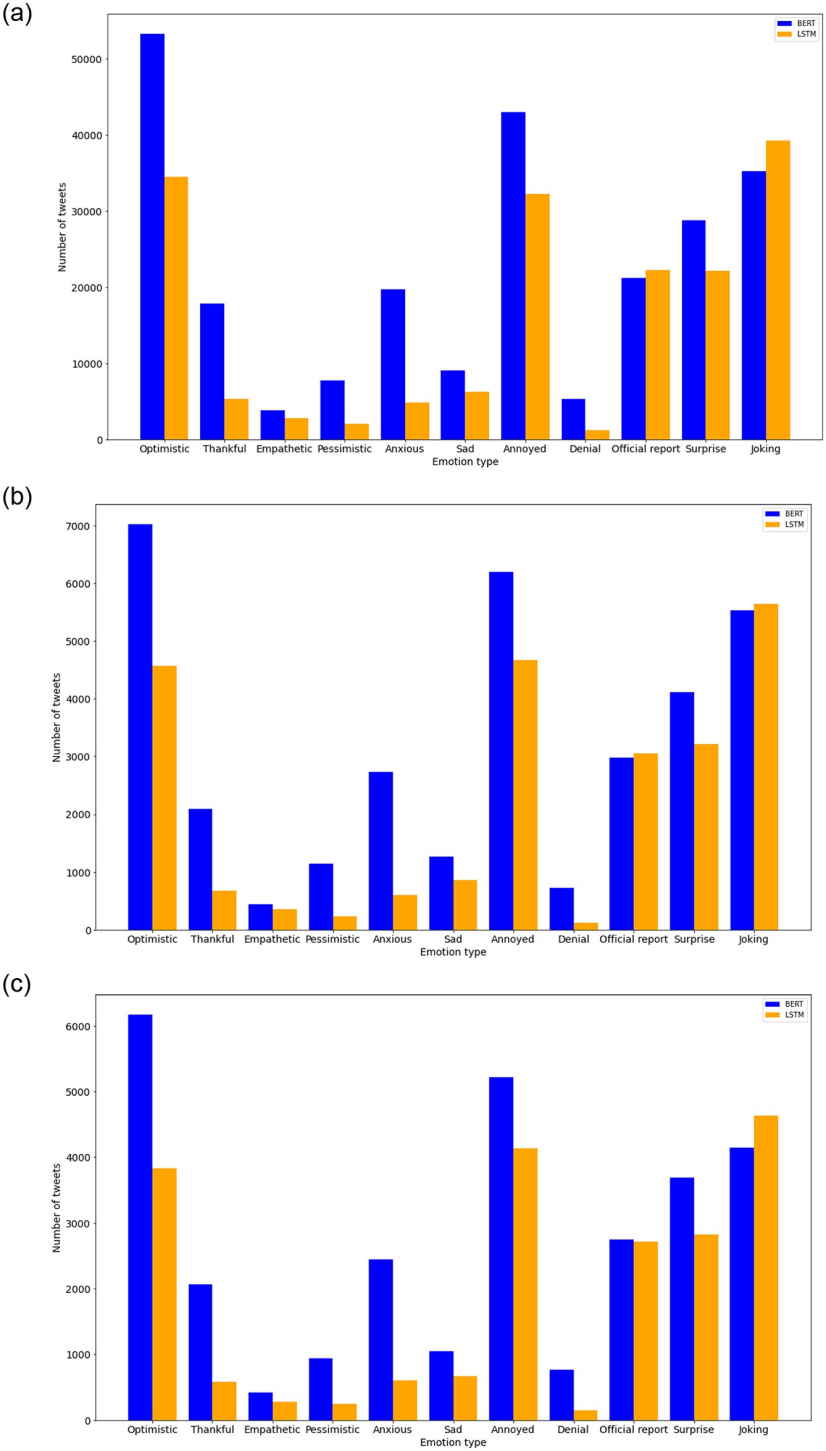
Distribution of sentiments predicted for the respective datasets by the LSTM and BERT models. (a) India. (b) Maharashtra. (c) Delhi.

In this section, we present results of the predictions of the COVID-19 tweets in India, Maharashtra, and Delhi treated as separate datasets. We note that in our Senwave dataset analysed in previous section has been used for training. The label “official report”, which is not a sentiment was included in the Senwave dataset, and hence we use it for prediction. Fig 5 presents distribution of sentiments predicted by the LSTM and BERT models for the respective datasets for the entire time-span of interest (March to September 2020). In the case of India (Panel a), we notice that the “optimistic”, “annoyed” and “joking” sentiments are the most expressed which also follows in the case of Maharashtra and Delhi (Panel b and c). We notice that the BERT model seems to capture more sentiments expressed when compared to the LSTM model, particularly the “optimistic”, “anxious”, and “annoyed” sentiments. We find that negative sentiments such as “pessimistic”, “anxious” and “sad” have been least expressed. We find “optimistic” sentiment as the most prominent sentiment which is followed by “annoyed” and “joking”. It is simpler to label “optimistic” and “thankful” as a positive sentiment, but it becomes increasing difficult when it comes to the sentiments “joking” and “surprise”, when context information is not available. Hence, it is insightful to review the sentiments such as “joking” and “surprise” in relation to other sentiments expressed. We find a similar trend in the case of Delhi and Maharashtra (Panel b and c) which are subsets of data from India (Panel a), the only major difference is the number of tweets which is significantly lower due to their respective population. Since the BERT model provided the best results for the training data, we provide the results by the BERT model henceforward.

**Fig 6:**
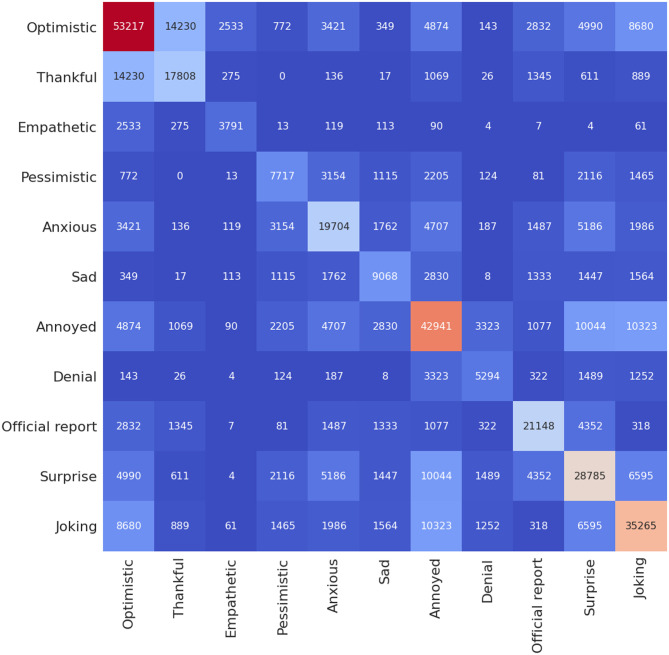
Heatmap showing number of occurrence of a given sentiment in relation to the rest of the sentiments for the India dataset using the BERT model.

**Fig 7:**
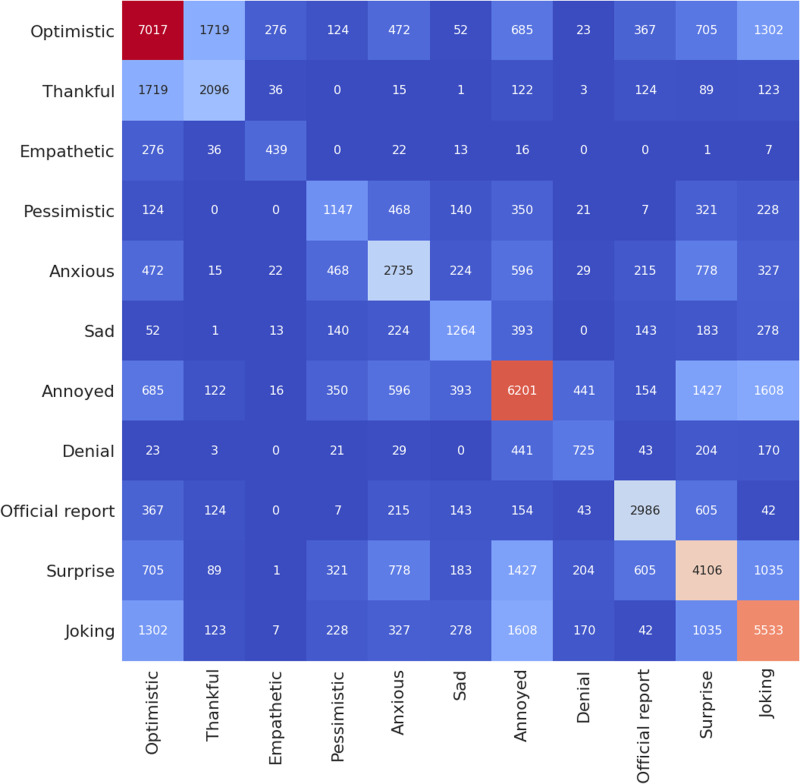
Heatmap showing number of occurrence of a given sentiment in relation to the rest of the sentiments for the Maharashtra dataset using the BERT model.

**Fig 8:**
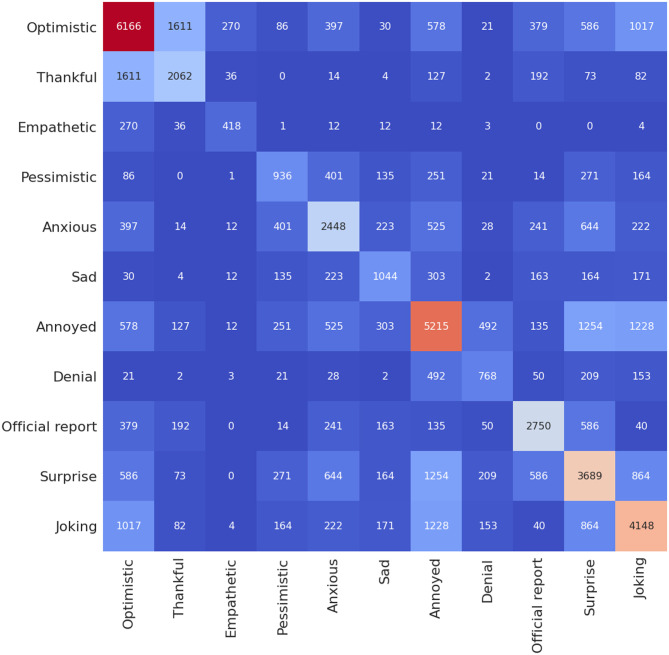
Heatmap showing number of occurrence of a given sentiment in relation to the rest of the sentiments for the Delhi dataset using the BERT model.

Next, we review the sentiments predicted using a heatmap to examine number of occurrence of a given sentiment in relation to the rest of the sentiments (Figs 6–8). These heatmaps essentially indicate how two sentiments have been expressed at once and provides more insights regarding the context of the negative and positive sentiments given in Fig 5. Note that in Fig 5, we found that the third most prominent sentiment has been “joking”; however, we were not sure if it is positive or negative sentiment. As shown in Fig 6, we find that most tweets that are associated with “joking” are either “optimistic” (8680) or “annoyed” (10323), and some are also “thankful” (889). A much lower portion (below 500) are either “empathetic” or “official report” while “joking”. Next, we review the sentiment “optimistic” and find that majority are either “thankful” (14230) or “joking” (8680). The “optimistic” tweets with negative sentiments are relatively minor, such as “pessimistic” (772), “denial” (143), and “sad” (349). Furthermore, a significant portion are also “empathetic” (2533). It is not common for one to make a statement that is optimistic and pessimistic at the same time; hence, this could be a wrong prediction by the model. However, it seems the model is making the right prediction when we look at the heatmap for the hand-labelled training datasets (Fig 4), where such combinations of sentiments have been labelled by experts. We show examples of tweets of this case and compare with those that are optimistic and thankful in Table 5.

**Table 5:** Selected example that show cases of tweets that are “optimistic” and also “pessimistic”, along with cases that are “optimistic” and also “thankful”.

Month	Tweet	Sentiment combination
May	“don’t care china only care indian covid19 news”	“optimistic—pessimistic”
August	“dear uddhav ji other than covid problem, what ever wrong is happening is not good for your government”	“optimistic—pessimistic”
September	“sir, plz look in have benefits to sr citizen.”	“optimistic—pessimistic”
April	“thank you switzerland smiling face with smiling eyes especially zermatt for showing” solidarity for india flag”	“optimistic—thankful”
May	“big thanks to the cfpc.…thoroughly enjoyed it!”	“optimistic—thankful”
June	“doctors, activists urge pm to promote plant-based diet—india news—times of india”	“optimistic—thankful”

Figs 7 and 8 show the number of occurrence of a given sentiment in relation to the rest of the sentiments for the case of Maharashtra and Delhi, which follows a similar pattern when compared to case of India (Fig 6)). We note that the Senwave dataset which shows tweets worldwide (Fig 4) follow a similar pattern than the case in Indian datasets when it comes to sentiments such as “joking” and being “optimistic” or “joking” and “annoyed”. Senwave dataset also features cases of being optimistic and pessimistic at the same time (226 cases). This could be due to sentiments expressed in two opposing sentences in a tweet. We can infer that since such patterns are part of the training data, it cannot be an error in predictions when looking at the Indian datasets.

**Fig 9:**
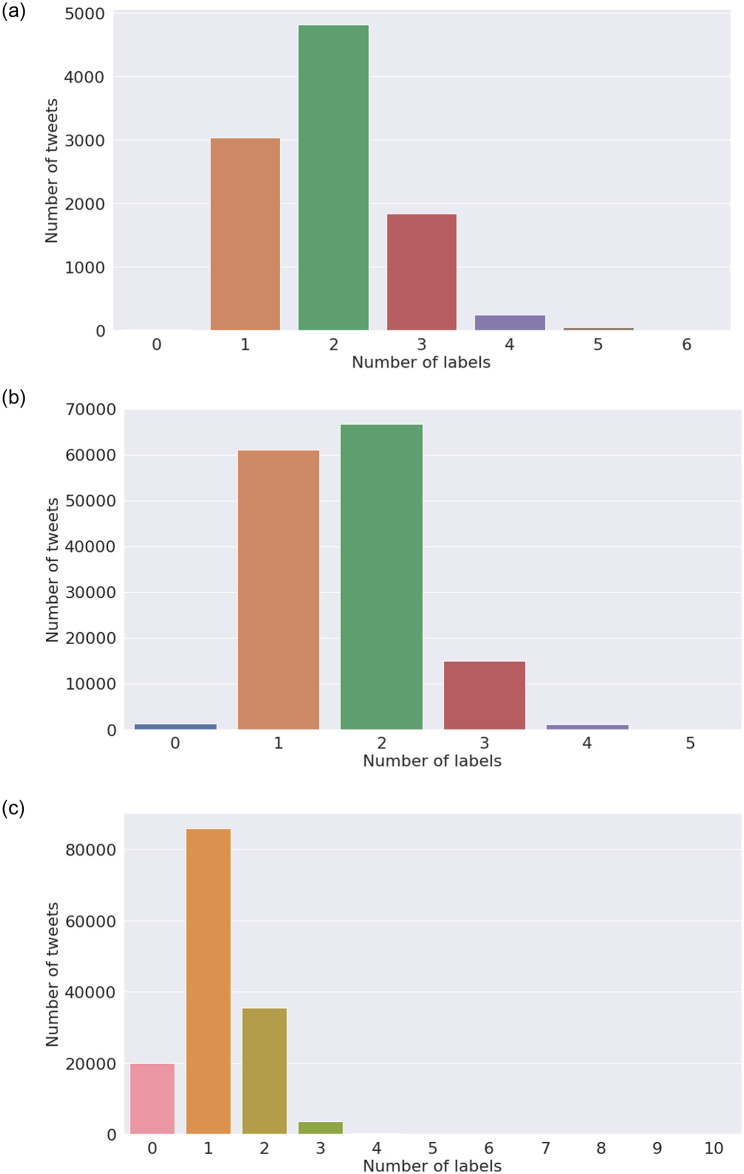
Distribution of tweets with number of combination of sentiment types (labels) for Senwave training dataset, and predictions by BERT and LSTM models. (a) Senwave hand-labelled sentiments (Worldwide). (b) Predicted (India) using BERT. (c) Predicted (India) using LSTM.

Fig 9 provides a visualisation of the distribution of tweets with number of combination sentiments. We find that around 60% of the tweets have a singular sentiment. Moreover, about 25% of the tweets have two sentiment attached them and 14% have no sentiment attached to them. Furthermore, a small number of tweets have 3 or more emotions attached to them which indicates that much often, people do not show multiple emotions at the same time.

**Fig 10:**
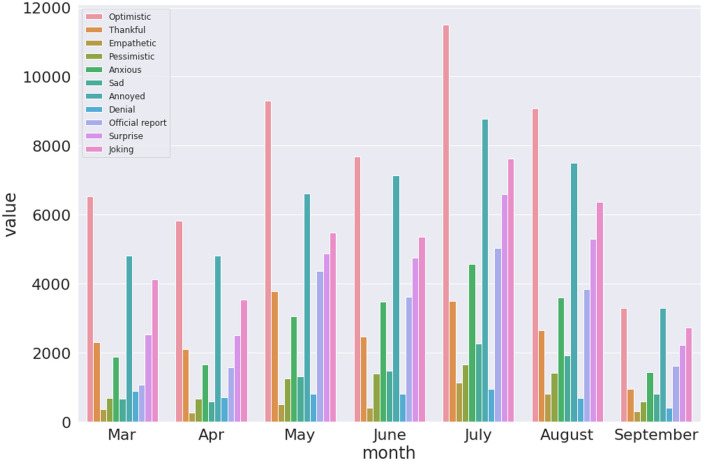
Monthly COVID-19 sentiments in India using the BERT model.

**Fig 11:**
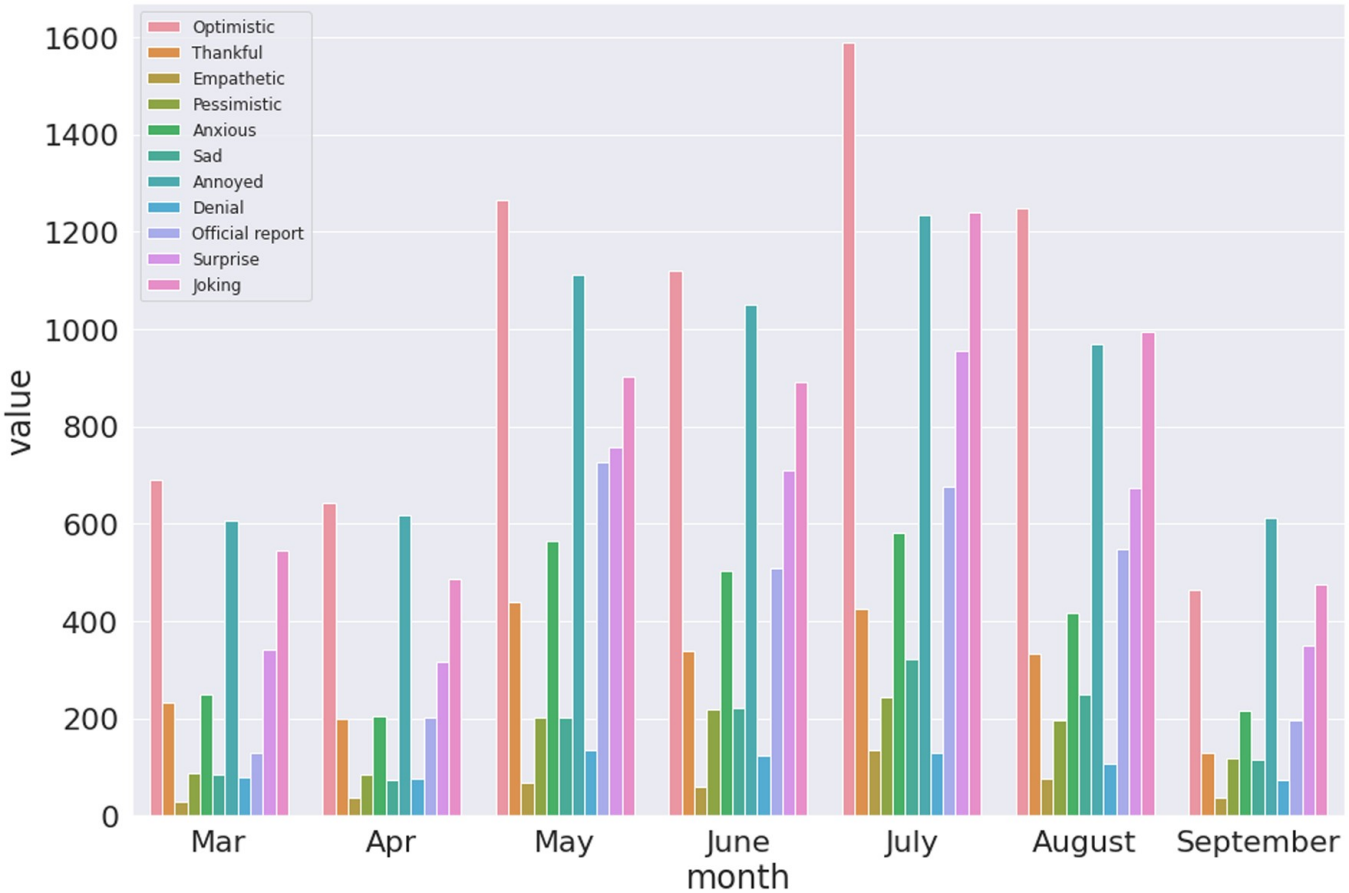
Monthly COVID-19 sentiments in Maharashtra using the BERT model.

**Fig 12:**
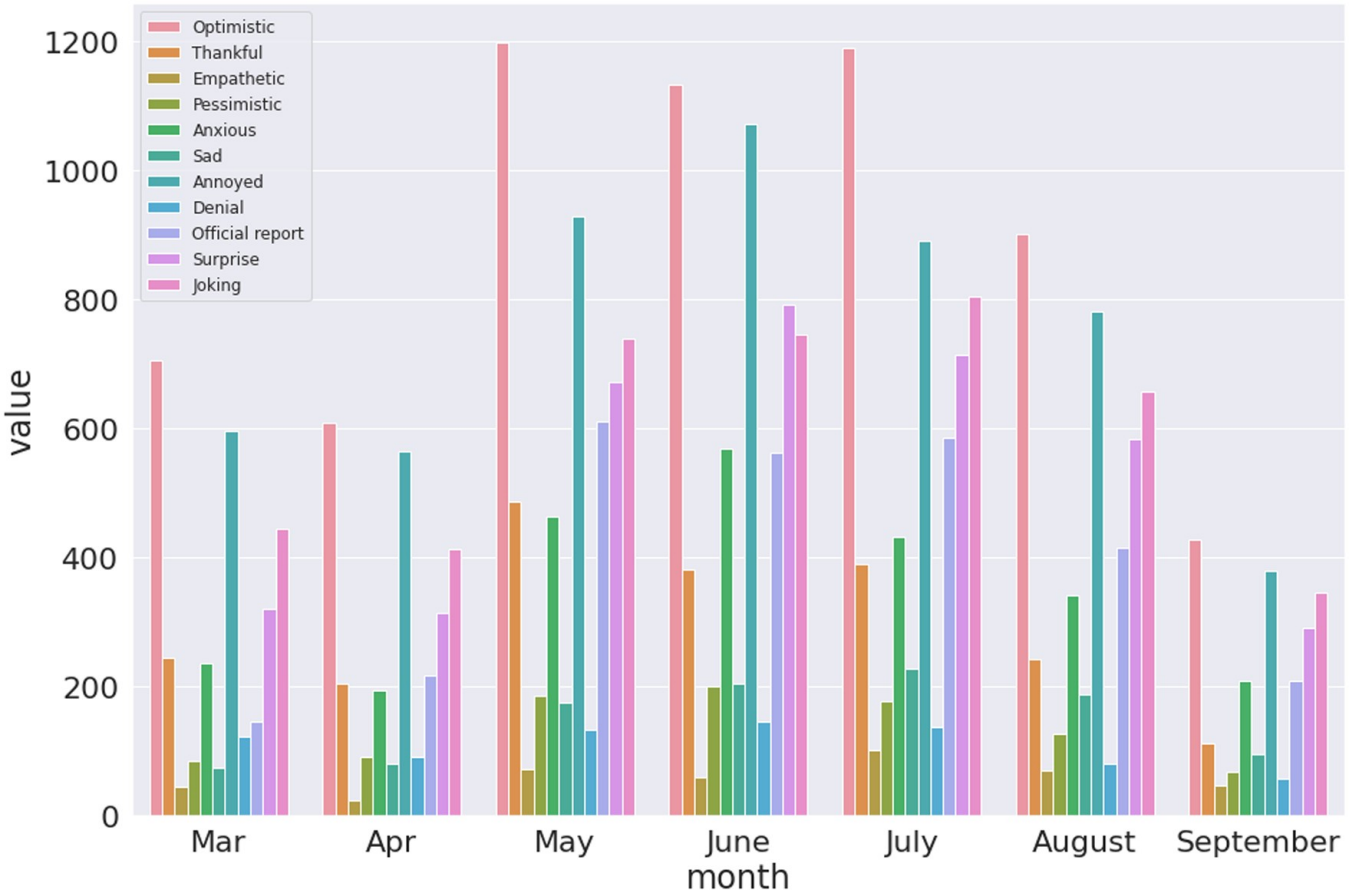
Monthly COVID-19 sentiments in Delhi using the BERT model.

Finally, we present results that visualises the trend of sentiments expressed over time in the three datasets. This is one of the key findings which can enable a understanding of the reaction in terms of emotions expressed by the population given rise and fall of COVID-19 novel cases as shown in Fig 2. Figs 10–12 presents visualisation of the monthly sentiments of India, Maharashtra and Delhi, respectively.

## 5 Discussion

Humans can express more than one sentiment at a time; however, there are variations in the number of sentiments that can be expressed by facial expressions [85], when compared to spoken or written emotions such as tweets. As shown in Fig 9, majority of the tweets have one sentiment, both in hand-labelled (Panel a) and predicted datasets (Panel b). This is followed by two sentiments, while a minority have 3 sentiments. We note that there is a significant portion of tweets with no sentiments and there are no tweets of more than three sentiments at a time. The study of emotions with technology has gained much interested in last decade which gave much progress in understanding sentiments. Trampe et al. [86] presented a study of everyday emotional experiences through an experience sampling smartphone application that monitored real-time emotions of more than 11,000 participants and found the group experienced at least one emotion 90% of the time with joy as the the most frequent emotion, followed by love and anxiety. The type of emotion would be highly dependent on the study region which featured Europeans with majority French participants. Cowen and Keltner reported [87] twenty seven distinct categories of emotions bridged by continuous gradients by data from emotionally evocative short videos with varying situational content. These studies contributed to better understanding of emotions given historical viewpoints about context and definitions of emotions and associations between them [88–91]. However, we did not find any study that reviews the number of emotions that can be expressed at a time in relation to catastrophic events that keep changing with time, such as the rise of COVID-19 cases.

We revisit the case of Indian dataset (Fig 10), where the monthly tweets did not sharply increase with number of novel cases (Fig 2) with a nationwide peak of novel cases (Fig 2, Panel a). The number of tweets gradually increased with peak of tweets in July (Fig 2, Panel a). When India had a peak of novel cases, we found that the number of tweets significantly lowered. Hence, people have been alarmed by rising cases, but a month before the peak of novel cases was seen, the tweets were reduced. Moreover, we find that the “optimistic”, “annoyed” and “joking” tweets are mostly dominating the monthly tweets for India (Fig 10) and Maharashtra (Fig 11), with a mix of annoyed sentiments in case of Delhi (Fig 12). There is significantly lower number of negative sentiments for the respective datasets (Figs 10–12).

We note that a limitation of the framework is due to the Senwave training data which considered tweets worldwide during COVID-19 by a group of experts; however, there can be limitations on how experts perceive humour in different regions. Humour is expressed differently in different cultural groups, i.e. a tweet that may be humorous in USA may not be taken as humorous in India due to cultural and language dimensions. There are several studies about the effect of humour in changing cultural or regional settings [92–94]. A good example is that in orthodox Chinese literature, humour was not expressed due to religious taboo in ancient Buddhism which later eased with Zen Buddhism [95, 96]; however, the Hindu literature had a different or eased attitude towards humour as expressed in ancient Hindu texts [97]. Although historic textual composition cannot be related to how tweets are expressed in India, it is important to note the cultural and language differences in how humour has been expressed.

Another limitation of the study is regarding the uncertainty of the data. Our results are based on the tweets expressed; however, we note that only a small fraction of the population generally express their views on Twitter. Moreover, we need to be aware that due to restrictions on freedom of speech and political biases, not everyone can express themselves freely. Social media networks have been active in monitoring how people express themselves in order to limit the rise of anti-vaccine sentiments. Furthermore, our framework’s training data is based on tweets expressed worldwide, whereas the test data is based on tweets from India during the pandemic. There is a large language diversity in India and at times, people express themselves with a combination of languages with geographic and culture specific jargon. Although, we convert them to English, certain limitations in capturing the context will exist.

We note that there has not been much work in uncertainty quantification for the predictions and there are different level of uncertainties, particularly in model parameters and data. We note that the training data is hand-labelled, and at times two or three sentiments have been expressed at once. This could be something open to interpretation by experts as it is hard to formally detect more than one sentiment from a tweet of only thirty words. Hence, the expert labelled training dataset adds to uncertainty in model predictions. In future work, Bayesian deep learning can provide a way to address uncertainty in model predictions [98–100].

## 6 Conclusions

We presented a study with novel deep learning models for sentimental analysis during the rise of COVID-19 infections. We selected tweets from India as our case study and reviewed tweets from specific regions that included Maharastra and Delhi. We took advantage of COVID-19 dataset of 10,000 hand-labelled tweets for training the respective deep learning models. Our investigation revealed that majority of the tweets have been “optimistic”, “annoyed” and “joking” that expresses optimism, fear and uncertainty during the rise of the COVID-19 cases in India. The number of tweets significantly lowered towards the peak of new cases. Furthermore, the optimistic, annoyed and joking tweets mostly dominated the monthly tweets with much lower number of negative sentiments expressed. We found that most tweets that have been associated with “joking” were either “optimistic” or “annoyed”, and minority of them were also “thankful”. In terms of the “annoyed” sentiments in tweets, mostly were either “surprised” or “joking”. These predictions generally indicate that although the majority have been optimistic, a significant group of population has been annoyed towards the way the pandemic was handled by the authorities. The major contribution of the paper is the framework which provides sentiment analysis in a population given the rise of the COVID-19 cases. The framework can be used by officials for better COVID-19 management through policies and projects, such as support for depression and mental health issues.

Future work can use the framework for different regions, countries, ethnic and social groups to understand their behaviour given multiple peaks of novel cases. The framework can be extended to understand reactions towards vaccinations with the rise of anti-vaccine sentiments given fear, insecurity and unpredictability of COVID-19. Finally, the framework can incorporate topic modelling with sentiment analysis which can provide more details for emerging topics during the rise of COVID-19 cases in relation to various government protocols such as lock-downs and vaccination plans.
